# Energy Sprawl Is the Largest Driver of Land Use Change in United States

**DOI:** 10.1371/journal.pone.0162269

**Published:** 2016-09-08

**Authors:** Anne M. Trainor, Robert I. McDonald, Joseph Fargione

**Affiliations:** 1 School of Forestry and Environmental Studies, Yale University, New Haven, Connecticut, United States of America; 2 Global Cities Program, The Nature Conservancy, Arlington, Virginia, United States of America; 3 North America Region, The Nature Conservancy, Minneapolis, Minnesota, United States of America; Clemson University, UNITED STATES

## Abstract

Energy production in the United States for domestic use and export is predicted to rise 27% by 2040. We quantify projected energy sprawl (new land required for energy production) in the United States through 2040. Over 200,000 km^2^ of additional land area will be directly impacted by energy development. When spacing requirements are included, over 800,000 km^2^ of additional land area will be affected by energy development, an area greater than the size of Texas. This pace of development in the United States is more than double the historic rate of urban and residential development, which has been the greatest driver of conversion in the United States since 1970, and is higher than projections for future land use change from residential development or agriculture. New technology now places 1.3 million km^2^ that had not previously experienced oil and gas development at risk of development for unconventional oil and gas. Renewable energy production can be sustained indefinitely on the same land base, while extractive energy must continually drill and mine new areas to sustain production. We calculated the number of years required for fossil energy production to expand to cover the same area as renewables, if both were to produce the same amount of energy each year. The land required for coal production would grow to equal or exceed that of wind, solar and geothermal energy within 2–31 years. In contrast, it would take hundreds of years for oil production to have the same energy sprawl as biofuels. Meeting energy demands while conserving nature will require increased energy conservation, in addition to distributed renewable energy and appropriate siting and mitigation.

## Introduction

By 2040, energy produced in the U.S. for domestic use and export is predicted to rise 27% to support both domestic and international demand [1]. The challenge of meeting energy demands while minimizing damaging climate change is widely recognized [2,3], but there is an additional challenge that also warrants attention–the land use implications of growing energy demand. The growing land use footprint of energy development, termed ‘energy sprawl,’ will likely cause significant habitat loss and fragmentation with associated impacts to biodiversity and ecosystem services [4–7]. However, the land use implications of future energy development are not well understood.

Here we use Energy Information Administration (EIA) analyses to estimate land area that will be newly required for energy production in the United States through 2040 [1]. Ours is the first analysis to take into account the ongoing and projected unconventional oil and gas development made possible by recent technological advances (i.e., hydraulic fracturing and directional drilling) [8–10]. This is important because by 2040, unconventional gas production in the United States is expected to double and will account for nearly 85% of all US natural gas production [1].

We also introduce a new concept, the “time to land use equivalency,” for renewable versus extractive (fossil fuel and nuclear) energy sources. Renewable energy can use the same land year after year [11,12], but extractive energy depletes local resources and, to sustain production, must continually drill and mine new areas [13–15]. Consequently, to produce an equal amount of energy, land requirements for fossil fuel and nuclear energy sources will, given enough time, equal and then exceed the land requirements for renewables. We calculate the number of years required to hit this land use equivalency threshold.

## Methods

We investigated the land use implications of EIA’s four different energy production scenarios from 2012 to 2040 (Table 1): 1) Reference, 2) Low Renewable Technology Cost, 3) High Oil and Gas Resources, and 4) Greenhouse Gas Tax. These scenarios represent a diverse range of plausible energy futures, and are evaluated in place of a single prediction, which would be highly uncertain given the dynamic nature of energy markets. The *Reference* scenario assumes “business as usual,” that is, that current policies continue to be implemented. The *Low Renewable Technology Cost* scenario assumes lower cost (20% below the Reference scenario assumptions) for new non-hydropower renewable electricity. Further, this scenario takes into account the Renewable Fuels Standard from the Energy Independence and Security Act (EISA) of 2007, which mandates the use of 36 billion gallons of renewable fuel by 2022 (the Reference case assumes that EPA continues to largely waive requirements for cellulosic and advanced biofuels). The *High Oil and Gas Resources* scenario forecasts greater production of technologically recoverable oil and natural gas resources in the US, such that production nearly doubles by 2040. The *Greenhouse Gas Tax* scenario assumes that Greenhouse Gases (GHG) are taxed at a rate of $10 per metric ton of CO_2_ equivalent, which discourages coal production and encourages less GHG-intensive energy sources.

**Table 1:** Proportion of cumulative energy produced from 2012 to 2040 in the United States for four scenarios.

Energy Product	Energy Source		Reference	Low cost renewable	High resource	GHG Tax
Electricity	Nuclear		12.4%	12.4%	11.6%	13.5%
	Coal		32.9%	32.5%	27.0%	29.2%
	Natural Gas	Shale Gas	22.9%	22.7%	27.1%	23.5%
		Tight Gas	11.8%	11.8%	14.3%	12.3%
		Coalbed Methane	3.1%	3.1%	2.7%	3.3%
	Renewables	Conventional	9.2%	9.2%	10.6%	9.7%
		Wind	2.6%	3.0%	2.3%	3.1%
		Geothermal	0.5%	0.5%	0.4%	0.6%
		Solar Photovoltaic	0.3%	0.5%	0.2%	0.5%
		Hydropower	4.1%	4.2%	3.9%	4.4%
		Solar Thermal	0.038%	0.038%	0.036%	0.040%
		Biomass	0.004%	0.011%	0.001%	0.005%
		**Total Kwhrs**	**204,917**	**204,806**	**216,603**	**195,351**
Liquid Fuel	Oil	Tight Oil	47%	46%	62%	47%
		Conventional	45%	44%	33%	45%
	Biofuels		9%	10%	5%	9%
		**Total Kwhrs**	**28,597**	**29,180**	**42,851**	**28,570**

Under each scenario, we calculated the amount of ‘new land area’ that would be required to meet future energy demands. New land area is defined as land area not currently used for energy production. These estimates account not only for the increasing rate of energy production, but also for the new development required to maintain production in the face of depletion of existing mines and wells.

Because the EIA specifies forecasts for a wide range of energy production categories (e.g., imports vs exports, onshore vs offshore, conventional vs various unconventional technologies, and utility vs end-user), we were able to constrain our analysis to the on-shore, domestic energy production relevant to land use impacts within the United States. Specifically, we considered energy produced in the United States, including energy exported out of the United States. Imported energy and offshore energy production were omitted from the analysis. Furthermore, the relatively small percentage (<10%) of end-use generation of electricity (firms or individuals that generate energy for their own consumption, also known as distributed energy, i.e., roof top solar [www.eia.gov]) was excluded because it generally occurs within the existing built environment and does not require development of new lands. Our analysis does not extend to the source materials used to construct energy infrastructure, whose mining and extraction would have an additional footprint, but which are sourced globally and so extend beyond the bounds of this analysis [16].

For each type of energy production, we compiled land requirements based on published literature, permitting agencies, aerial imagery, and other public sources (e.g., [16–22], S1 Table). When sufficient data were available (> 10 estimates), the median value was used as a ‘representative’ value. To account for multiple uncertainties stemming from variability among producers and states, we provide a high and low estimate of energy sprawl with each scenario by using the 25^th^ and 75^th^ quantiles to estimate low and high impacts, respectively. When insufficient data were available after an extensive literature search, we selected values from the limited available resources to characterize representative, low, and high land use impacts.

We estimated land area requirements in two ways, one based on the direct footprint and one based on total area required. The direct footprint included, for example, the area cleared for reservoirs of hydroelectric dams, well pads, mines, associated roads, pipelines, and wastewater storage. The total area required is often greater than the directly impacted area and accounts for any spacing requirements. For example, wind turbines are commonly spaced 4 rotor diameter widths apart from side to side and 10 rotor widths apart from front to back, to minimize turbulence that affects energy production [23]. Thus, only about 3% of a wind development is directly impacted by turbines, roads and operations facilities, but the total area required includes the land in between the turbines [5,24]. Similarly, there are technical and regulatory limitations on how closely oil and gas wells can be spaced together, and the total area required for development includes the land between the wells [25,26]. Given the fragmenting effects of turbines and oil and gas wells on animal movement, reproduction and survival, there are known ecological impacts on the entirety of this ‘total area required’ [7,20,27,28]. Indeed, our estimate of the total area required is likely a conservative indicator of the area experiencing ecological impacts, which can extend beyond the project boundary of any particular energy development [13,29]. Hereafter, we refer to the total area required as the landscape impact, since it conservatively characterizes the impact to landscapes.

### Renewable Electricity

The energy production from wind, solar, hydroelectric, geothermal and bioelectricity is predicted to increase through 2040. The new land required for renewable energy depends on the projected maximum annual production rather than cumulative production, so we compared 2040 with 2011 for each renewable energy sector to calculate the amount of new annual energy production and associated new land use.

The footprint of hydroelectric power was determined by calculating the median land area inundated with water above 47 hydroelectric dams randomly selected from the National Hydrography Dataset (NHD; [30]). The area for each dam was then scaled by annual energy production to estimate the footprint required per unit energy produced [31]. For wind energy, we relied upon a comprehensive study of land impacts for 161 wind projects throughout the US [32].

For each renewable energy source, we estimated an average capacity factor, which specifies the percentage of energy produced compared to a facility operating continuously at full capacity (http://energy.gov/eere; S1 and S2 Appendices). For example, modern land-based wind turbines commonly have a 2.5 MW capacity, but only produce this amount of energy when the wind is blowing fast enough, which averages out to 33% of the time. Land use impacts depend on installed capacity, so we used capacity factors to determine how much installed capacity would be needed to generate the energy production predicted by EIA scenarios.

### Biofuels

Biofuels include ethanol, biodiesel, and other liquid fuels made from biomass. The EIA estimates biofuel production from four categories of feedstock: 1) corn, 2) soybean, 3) sugarcane, and 4) other biomass. As with renewable electricity, maximum production volumes between 2012 and 2040 were compared with production in 2011 to determine the amount of additional land that will be required for each type of biofuel. For corn and soybean, we estimated crop yields using USDA projected long-term trends (extrapolated to 2040) [33,34]. We assumed that the “other biomass” category was supplied by dedicated energy crops such as switchgrass (*Panicum virgatum*), Miscanthus (*Miscanthus giganteus*) and short rotation woody crops [35,36]. Since these feedstocks are not currently being used for commercial biofuel production in the US [1], we used literature values for yields to estimate their spatial impacts (S2 Table).

Biofuels made from corn and soybean produce co-products that are fed to livestock, displacing the need for other livestock feed, and reducing the amount of additional land required to produce biofuels [37]. We allocated land use to biofuels versus co-products based on energy content [38]. Note that our methodology tracks the direct land-use needs of biofuel production, and does not consider indirect effects on land-use via agricultural commodity markets. For example, if a soybean field in the U.S. is switched to corn to make ethanol, then soybean production may expand elsewhere either domestically or internationally. Modeling land use effects as mediated by global agricultural trade is beyond the scope of this paper, but is an active area of research [39].

### Mined Energy Sources

For energy sources associated with mining (coal and nuclear), we used EIA’s cumulative production from 2012 to 2040 to estimate land use impacts. For each energy source, we estimated the areal impact of mining and plant operations [6]. For nuclear energy, we also estimated the areal impact of waste storage.

For coal, EIA estimates domestic production in three geographic regions (Appalachia, Interior, and West). We assumed that the current proportion of coal mined on the surface versus underground in each region is maintained through 2040 [1]. Area impacted due to surface mining activities varies by region [1]. Specifically, the footprint of coal mining is larger in the West’s open pit mines (~ 515 ha/million short tons) than it is in Appalachian’s mountaintop removal mines (~212 ha/million short tons). In contrast, there was little geographic variation in land use impacts for underground coal mining, allowing us to use a single nationwide estimate.

### Drilled Energy Resources

For energy sources associated with drilling, we used EIA’s cumulative production from 2012 to 2040 to estimate land use impacts. Drilled resources include both oil (conventional and tight oil) and natural gas (conventional, shale gas, tight gas, and coalbed methane). For each energy type, we estimated the number of wells drilled through 2040 and the amount of area required per well and multiplied these numbers to estimate total land use. For each scenario, EIA estimates the total number of wells drilled each year, but does not specify how many of these wells are attributable to each type of production. To address this, we developed an approach that is internally consistent with EIA’s scenarios in terms of the total number of wells, while still allowing us to quantify the land use associated with each energy type. To meet these two requirements, we produced our own estimates of the number of new wells that would be required to meet EIA’s projected production for each energy type, and used the proportion of wells from our estimates for each energy type to apportion EIA’s total well numbers.

To produce our estimates of well numbers, we used average annual production values and an average well abandonment rate to calculate the number of new wells required to achieve projected production levels each year. For both conventional and unconventional sources, well abandonment rates were assumed to be the inverse of well lifetime [40,41]. For unconventional wells, average annual production was calculated as projected production during entire well lifetime, divided by well lifetime in years. Projected production was an average of EIA estimates across all unconventional plays (similar oil and gas accumulations sharing geologic and geographic properties), weighted by the recoverable resource in each play. EIA did not estimate total production during entire well lifetime for conventional wells. To calculate average annual production for conventional wells, we divided the total annual production by the number of producing wells for each year between 1995 and 2005 (before the proliferation of unconventional wells). We used our estimated well numbers to apportion EIA’s total well projections among energy types.

We calculated the direct footprint and landscape impacts for each well type. The direct footprint included the well pad (including wastewater holding ponds and staging areas for hydrological fracturing equipment) and roads and pipelines. For the landscape impact of unconventional drilling, we used a weighted average of EIA’s well spacing values (wells/miles^2^), which are estimated for each play. To estimate spacing requirements for conventional oil and gas, we used spacing rules codified in state policy that require no more than one well for every 16 acres [42–44].

### Land use efficiency

For each energy sector, we derive an empirical estimate of the amount of land required to produce a given amount of energy in a given year, termed land use efficiency. This is expressed as the square kilometers required for each TWhr produced (km^2^/TWhr). For wind and drilled resources, we measure landscape impact in addition to the direct footprint, which includes spacing requirements.

### Time to land use equivalency

**Fig 1:**
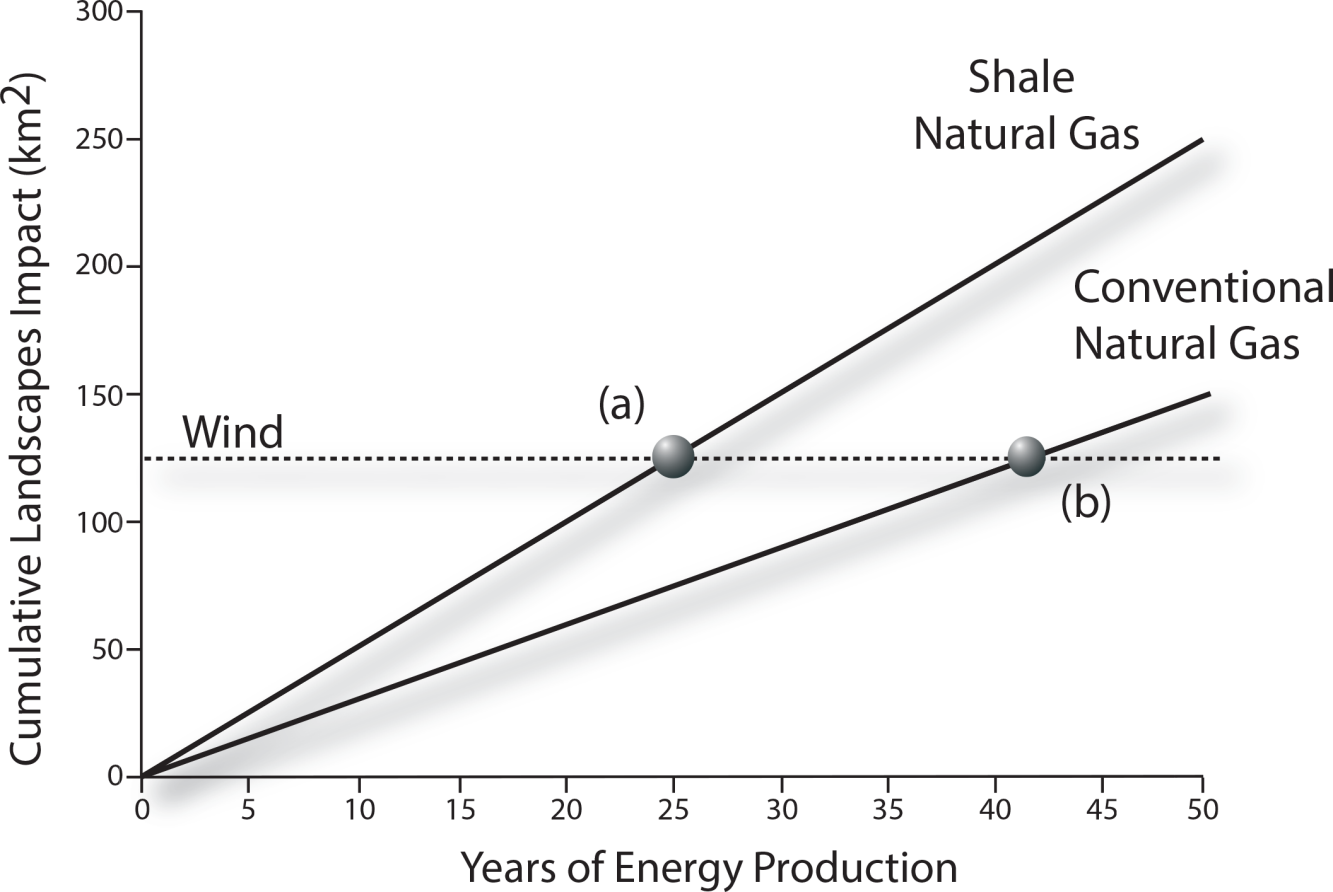
Schematic explaining time to land use equivalency for renewable and extractive energy sources. The cumulative land required to produce 1 TWhr/year is shown for three energy sources. Renewable energy sources can reuse the same land every year, such that there is no increase in cumulative land required. In contrast, the land required to acquire extractive energy sources expands every year. Point (A) shows the time to land use equivalency between wind and shale natural gas (25 years) and point (B) shows the time to land use equivalency between wind and conventional gas (44 years). Both examples are calculated for landscape impacts.

Because renewable energy production can re-use the same land footprint every year and extractive energy sources must expand as wells and mines are depleted, a direct comparison of land use requirements can only be made over a particular time horizon with a specified annual energy production schedule. Because renewable energy sources have lower energy density than extractive sources, they generally require much more land to produce energy, when measured over a single year. However, because renewables can re-use the same land, their cumulative energy production can increase every year without any increase in their cumulative land footprint. In contrast, extractive energy sources must expand their footprint to acquire additional resources as wells dry up and mines are depleted. Consequently, for a fixed amount of annual energy production, we can calculate the number of years it would take for extractive energy production to achieve the same land footprint as renewable energy for an equivalent of cumulative energy production (Fig 1). This approach complements alternative life-cycle assessment approaches that apply to fixed time horizons or assume particular temporal discounting rates, both of which can be contentious [45].

## Results

### Recent Energy Sprawl

**Fig 2:**
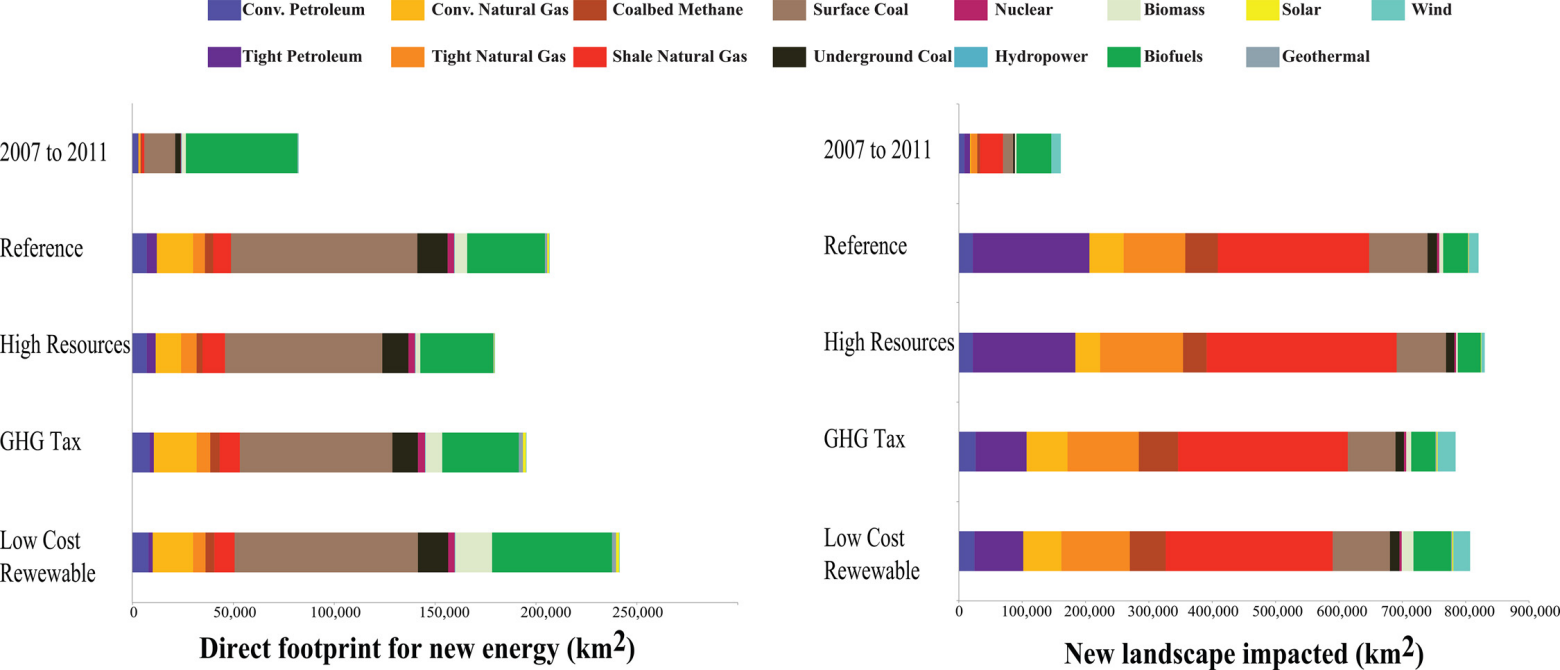
New land area impacted from energy development in the United States between 2007 and 2011 (top bars) and between 2012 and 2040 under four EIA scenarios. The left panel shows the direct footprint and the right panel shows landscape impacts, as defined in the text.

Between 2007 and 2011, the United States increased its energy production by 15% [1]. Over 82,000 km^2^ were directly impacted by new energy infrastructure, an area nearly the size of Maine (Fig 2 top row). The landscape impacts were nearly double, at 161,000 km^2^, due to the spacing requirements of wells and wind turbines.

Considering the direct footprint, biofuels accounted for two thirds of the energy sprawl (67%; 55,390 km^2^), despite comprising only 6% of total energy production. The remaining sources of the direct footprint from energy sprawl were 22% from coal, 7% from oil and natural gas, 3% from other renewables, and <1% from nuclear. Landscape impacts were led by oil and gas with 43% of the energy sprawl. This was followed by 34% from biofuels, 11% from coal, 9% from wind, 2% from other renewables, and < 1% from nuclear.

### Future Energy Sprawl

Based on EIA’s scenarios, energy sprawl in the United States will directly impact an additional 179,637 to 241,580 km^2^ by 2040 (Fig 2). Depending on our choice of an energy future, there is a roughly 62,000 km^2^ difference in the amount of land we would need to set aside. Regardless of the scenario, approximately half the direct footprint through 2040 will be associated with coal mining (43–52% of the total direct footprint). The western region (Arizona, Colorado, Montana, North Dakota, New Mexico, South Dakota, Utah, and Wyoming) will experience the greatest impacts from coal mining (41,776 to 112,512 km^2^).

**Fig 3:**
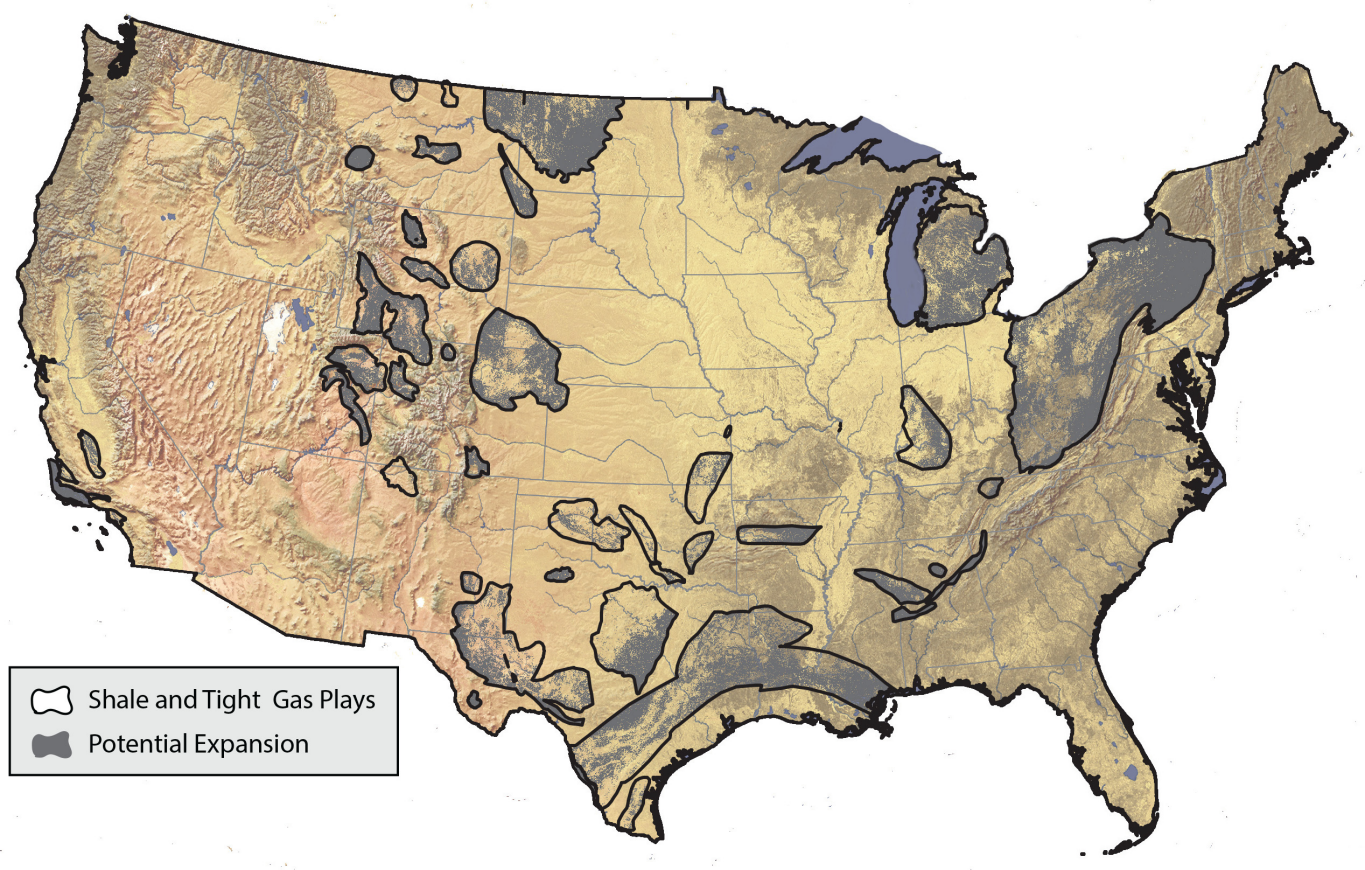
Areas at risk of development from unconventional oil and gas drilling that had previously not experienced conventional oil and gas drilling. We included EIA’s shale and tight oil and gas plays, but removed areas of existing conventional oil and gas development, based on historical oil and gas exploration and production wells drilled between 1900 and 2006 in the United States [73]. We identified and removed the 666,009 km^2^ that have or had well activities in the past.

Oil and natural gas will contribute 25–33% of the direct footprint from energy sprawl (57,737 to 64,309 km^2^) but comprise a dominant 73–83% of the landscape impacts (690,911 to 589,882 km^2^). Until recently, the majority of natural gas production was from conventional gas. However, between 2007 and 2011, approximately 70% of natural gas production was from unconventional sources and this is predicted to increase to 85% by 2040. This rapid expansion of unconventional gas will have a landscape impact of 238,961 km^2^, in our reference scenario. The unconventional gas revolution is significantly expanding the geographies that will be impacted by natural gas development. Approximately 1.3 million km^2^ that had never experienced conventional oil and gas drilling are now at risk of being drilled for unconventional oil and gas (Fig 3).

### Land-Use Efficiency

Land use efficiency varies greatly across energy production types, from 0.13 km^2^/TWhr for nuclear to 809 km^2^/TWhr for biomass (Table 2). Among fossil fuels, surface coal has the greatest direct energy sprawl (8.2 km^2^/TWhr). The relative efficiency of conventional and unconventional natural gas depends on whether the direct or the landscape energy sprawl is considered. The direct footprints of unconventional oil and natural gas production are smaller than their conventional counterparts (24% smaller for oil and 57% smaller for gas). But the landscape impact of unconventional oil and natural gas are bigger than their conventional counterparts (96% bigger for oil and 95% bigger for gas).

**Table 2:** Range of land use efficiency for each energy source.

			Land-use Efficiency (km^2^/TWhr)		
Energy Product	Energy Source		Area of Direct footprint (lower-upper estimates)		Landscape-level Impact*
Electricity	Nuclear		0.13	(0.02–0.24)	0.13
	Natural Gas	Shale Gas	0.19	(0.12–0.48)	5.08
		Tight Gas	0.24	(0.13–0.89)	4.01
		Coalbed Methane	0.63	(0.28–0.81)	8.11
		Conventional	0.95	(0.82–0.951)	2.86
	Coal	Underground	0.64	(0.24–1.51)	0.64
		Surface	8.19	(4.69–16.42)	8.19
	Renewables	Wind	1.31	(0.34–1.37)	126.92
		Geothermal	5.14	(2.14–10.96)	5.14
		Solar Photovoltaic	15.01	(12.30–16.97)	15.01
		Hydropower	16.86	(6.45–86.95)	16.86
		Solar Thermal	19.25	(12.97–27.98)	19.25
		Biomass	809.74	(557.93–1254.028)	809.74
					
Liquid Fuel	Oil	Tight Oil	0.38	(0.23–0.88)	8.19
		Conventional	0.56	(0.48–0.66)	2.86
	Biofuel	Corn	236.59	(192.69–259.00)	236.59
		Sugarcane	274.49	(229.24–342.05)	274.49
		Soybean	295.91	(235.54–313.33)	295.91
		Cellulose	565.39	(125.67–826.49)	565.39

* Energy sources without spacing requirement have the same value for direct and landscape-level impacts.

Per unit energy, renewable energy generally has a greater direct footprint than extractive energy. Wind infrastructure is the most land-use efficient (1.3 km^2^/TWhr) form of renewable energy, when considering direct footprint. However, when landscape impact is considered, wind becomes one of the least land-use efficient sources of electricity (126.9 km^2^/TWhr), second only to biomass (809.7 km^2^/TWhr) and biofuels (>200 km^2^/TWhr). Photovoltaic solar technology is slightly more efficient (15.0 km^2^/TWhr) than thermal solar technology (19.3 km^2^/TWhr).

### Time to Land Use Equivalency

The time to land use equivalency differed between electricity (Table 3) and liquid fuels (Table 4). The time to land use equivalency was generally less than 40 years for electricity production from renewables other than biomass (with the exception of the landscape impact of wind compared to conventional gas and underground coal). In contrast, when considering renewable liquid fuels, biofuels have land use impacts that would require hundreds of years for petroleum-based fuels to equal.

**Table 3:** Years to land use equivalency in electricity sector.

Direct Footprint							
	Extractives						
		Natural Gas				Coal	
Renewables	Nuclear	Conventional	Shale Gas	Tight Gas	Coalbed Methane	Underground	Surface
Wind	9.9	1.4	6.9	5.5	2.1	2.1	0.2
Geothermal	39.0	5.4	26.9	21.7	8.1	8.1	0.6
Solar Photovoltaic	114.0	15.8	78.5	63.5	23.7	23.6	1.8
Hydropower	128.0	17.7	88.2	71.3	26.6	26.5	2.1
Solar Thermal	146.1	20.2	100.7	81.4	30.4	30.2	2.4
Biomass	6149.0	850.8	4235.9	3423.2	1280.1	1272.5	98.9
Landscape impact							
	Extractives						
		Natural Gas				Coal	
Renewables	Nuclear	Conventional	Shale Gas	Tight Gas	Coalbed Methane	Underground	Surface
Geothermal	39.0	1.8	1.0	1.3	0.6	8.1	0.6
Solar Photovoltaic	114.0	5.2	3.0	3.7	1.9	23.6	1.8
Hydropower	128.0	5.9	3.3	4.2	2.1	26.5	2.1
Solar Thermal	146.1	6.7	3.8	4.8	2.4	30.2	2.4
Wind (Total area)	963.8	44.3	25.0	31.7	15.7	199.5	15.5
Biomass	6149.0	282.9	159.5	202.1	99.9	1272.5	98.9

The years to land use equivalency is the number of years of energy production for which the listed renewable and extractive energy sources have the same land use impact.

**Table 4:** Years to land use equivalency in liquid fuel sector.

Direct Footprint		
	Extractives	
Renewables	Conventional Oil	Tight Oil
Corn	420.7	628.1
Sugar Cane	488.1	728.7
Soybean	526.2	785.6
Cellulose	1005.3	1501.0
Landscape Impact		
	Extractives	
Renewables	Conventional Oil	Tight Oil
Corn	82.7	28.9
Sugar Cane	95.9	33.5
Soybean	103.4	36.1
Cellulose	197.5	69.1

The years to land use equivalency is the number of years of energy production for which the listed renewable and extractive energy sources have the same land use impact.

### Benefits of Energy Conservation

Our results indicate the importance of energy conservation to curtail energy sprawl. We find that every 1% of energy conservation would save roughly 2,000 km^2^ from being directly impacted with energy infrastructure by 2040 (Table 5). When the landscape impact of electricity is considered, over 8,000 km^2^ of impact could be avoided for every 1% conserved.

**Table 5:** Avoided km^2^ of energy sprawl from 1% energy conservation.

Type of conservation	Reference	Low Cost Renewables	High Resource	GHG Tax
Electricity direct footprint	1,569	1,735	1,329	1,480
Liquid fuel direct footprint	503	696	475	487
Total direct footprint	2,072	2,431	1,804	1,967
Electricity landscape impact	5,770	6,472	6,100	6,400
Liquid fuel landscape impact	2,444	1,609	2,201	1,451
Total landscape impact	8,214	8,081	8,301	7,851

## Discussion

To meet growing energy demands in the United States, roughly 200,000 km^2^ of the country will be directly impacted by energy development by 2040. Furthermore, when we include spacing requirements, over 800,000 km^2^ of landscapes will be impacted by energy development. This gives an average rate of direct land use change of 6,900 km^2^ yr^-1^. By comparison, between 1973 and 2000, developed areas in the United Sates (residential, urban, commercial) increased at a rate of 2,900 km^2^ yr^-1^ [46]. Looking forward, across multiple scenarios, maximum rates of land use change in the United States through 2051 were projected as 5,600 km^2^ yr^-1^ for cropland and 5,900 km^2^ yr^-1^ for developed areas [47]. In total, energy sprawl is causing land use change at rates higher than other major drivers, making it the largest driver of land use change in the United States. These results highlight the need for policies that safeguard biodiversity and ecosystem services against the cumulative impacts of energy development.

What do our results suggest about which sources of energy are desirable from a land use perspective? We conclude that all forms of energy production can have significant land use impacts, and that simply dictating particular forms of energy production is inadequate: safeguards related to siting, mitigation, and energy conservation and efficiency will be required.

Renewables have the advantage of being able to reuse the same land every year, which can compensate for the generally lower land use efficiency of renewable energy. For renewable electricity, the time to land use equivalency was generally several decades or shorter. Thus, over the likely lifetime of an energy development project, renewables are comparable to conventional energy from a land use perspective.

The notable exception is biofuels. When produced from dedicated energy crops, the land use efficiency of biofuels is so low that it would take hundreds of years of oil production to have an equivalent land use impact. Over two thirds of recent direct energy sprawl– 55,000 km^2^ from 2007–2011 –was due to biofuels expansion. This was spurred by the Energy Independence and Security Act of 2007, which mandated 136 billion liters [36 billion gallons] of biofuels by 2022 [1,48]. To meet the 2022 biofuel mandate with dedicated energy crops would require an additional 59,500 km^2^ of land for biofuels. In total, between 2007 and 2040, biofuels could impact an area larger than Virginia. This extensive direct footprint from energy sprawl is not compatible with habitat conservation goals. Our results suggest that fuel cell and electric vehicles powered by wind, solar, or nuclear would not only have lower greenhouse gas emissions [49], but also lower land use impact than biofuels.

At present, the cost and benefit of nuclear energy is being vigorously debated (i.e., [50,51–53]), We find that nuclear energy has, in addition to low greenhouse gas emissions [49,54], a small land use footprint. This suggests that technological solutions to issues hindering the expansion of nuclear power (including nuclear waste disposal, proliferation, and power plant failures) could yield land conservation benefits.

Unconventional oil and gas development is similar to conventional development from a land use perspective, but has allowed dramatic expansion of energy production into areas previously unsuitable for drilling [7,13]. Compared to conventional oil and gas, unconventional oil and gas have lower direct footprints because more wells can be drilled on each pad, but a higher landscape impact because the amount of energy extracted across each square km is lower. However, current estimates of the direct footprint are sensitive to assumptions about the productivity of each well, and there is debate as to how quickly production from unconventional wells will decline [8,13,55].

Because new technology makes previously unsuitable resources economically recoverable, unconventional development has opened up over 1.3 million km^2^ to potential development (Fig 3), and we expect up to 520,518 km^2^ of this landscape to be impacted by unconventional oil and gas development. The expansion of drilling into communities where it was previously unknown has resulted in a wide range of public concerns and regulatory responses [56–58]. This highlights the importance of ensuring that all forms of energy development avoid areas that are most sensitive to habitat loss and fragmentation, reuse existing rights of way and adequately fund compensatory offsite mitigation [59,60].

There are a variety of approaches to energy production that avoid and minimize energy sprawl. End-use production holds promise for reducing energy sprawl. Solar panels on buildings and other efforts to incorporate energy production into the built environment increase energy production without requiring the conversion of natural areas [61,62]. Similarly, energy production with large spacing requirements, such as wind energy and oil and gas production, can often be co-located with cropland such that no additional natural areas are impacted [63]. Infilling natural gas wells instead of extending into new areas could significantly reduction land disturbance and fragmentation [7]. Biofuels made from agricultural residues or wastes could substantially reduce land use impacts [64].

Energy conservation can help avoid energy sprawl. Efficiency standards for vehicles, appliances, and buildings can reduce overall emissions [65–67], especially when combined with policies that promote conservation [68]. Roughly 4% or 10,000 km^2^ of landscape impact would be avoided with every 1 mpg increase in the average fuel efficiency of the U.S. vehicle fleet [69]. Simple household electricity savings, such as switching to compact fluorescent bulbs, would save roughly 2.5% of total US electricity use, avoiding over 14,000 km^2^ of impact from energy sprawl by 2040 [70,71].

Even with substantial increases in end use generation and energy conservation, large areas will still be required for new energy development. Improved siting and mitigation practices are needed to avoid and compensate for impacts, taking into account the cumulative landscape impacts of projected energy development [59,60,72]. To meet energy demands while conserving nature will require the full suite of actions discussed here: conservation and efficiency in addition to distributed renewable energy and appropriate siting and mitigation.

## Supporting Information

S1 Appendix Guidance and notes on Navigating Energy sprawl Excel Document. Energy Sprawl is the Largest Driver of Land Use Change in United States. This word document provides detailed expiation on how to navigate the complex S2 Appendix excel file.
(DOCX)

S2 Appendix Excel file containing all the energy production estimates provided by Energy Information Administration, the calculations used to estimate footprint for each energy source, the conversion equations, and the approach used to estimate the total energy sprawl. (XLS)

S1 Table Estimated footprint and citations used to estimate the compact, representative, and broad spatial impact for each energy source. (DOCX)

S2 Table List of experimental studies used to estimate the representative spatial impact of “other biomass used to produce any liquid fuel.” (DOCX)
