# Supplementary material for: Energy Sprawl Is the Largest Driver of Land Use Change in United States
Source: PLoS One. 2016 Sep 8;11(9):e0162269. doi: 10.1371/journal.pone.0162269 (PMC5015902; doi:10.1371/journal.pone.0162269)
Supplement: S1 Appendix — Energy Sprawl is the Largest Driver of Land Use Change in United States. This word document provides detailed expiation on how to navigate the complex S2 Appendix excel file. (DOCX) [file pone.0162269.s001.docx]

**Guidance and notes on Navigating Energy sprawl Excel Document**

**Energy Sprawl is the Largest Driver of Land Use Change in United States**

FootprintEstimates Worksheet

This sheet provides information on the spatial requirements (direct footprint and landscape impact) for each energy sector in the original units for that sector. To account for variability in industry and geography we estimate representative and high and low values. These estimated values are linked to each sector’s calculations. Therefore, changing these values will adjust the final footprint estimates.

CoalImpacts_reps

This sheet calculates a representative estimate of coal impact for surface and below ground mining. Below is the description for each section:

Rows 3-13: Estimates of energy production provided by EIA.

Rows 15-23: Cumulative production from EIA

Rows 25-29: According to the EIA (https://www.eia.gov/coal/annual/pdf/table1.pdf), this is the proportion of each coal mine type.

Rows 31-37: The footprint, linked to FootprintEstimates worksheet

Rows 39-59: The estimated area of impact for cumulative production of coal for each scenario and type of coal mining, using this equation:

Area (ha) = EIA Cumulative Coal Production (million short tons) * % production for each type of coal mining * Footprint for that type of coal mining (ha/million short tons)

Rows 62-118: The calculations to convert impact (ha) and energy production (million short tons) into consistent units (km2/TWhr)

Row 122-133: Unit conversion of cumulative production in million short tons to TWhr

CoalImpacts_Bounds

This sheet calculates low and high estimates of coal impact for surface and below ground mining. Below is the description for each section:

Rows 3- 13: Estimates of energy production provided by EIA.

Rows 15-23: Cumulative production in million short tons

Rows 25-29: According to the EIA (https://www.eia.gov/coal/annual/pdf/table1.pdf), this is the proportion of each coal mines by type.

Rows 31-41: The footprint linked to FootprintEstimates worksheet (low and high estimates)

Rows 43-82: The estimated area of impact for cumulative production of Coal for each scenario and type of coal mining

Area (ha) = EIA Cumulative Coal Production (million short tons) * % production for each type of coal mining * Footprint for that type of coal mining (ha/million short tons)

Rows 83-108: The calculations to convert impact (ha) and energy production (million short tons) into consistent units (km2/TWhr)

Row 113-125: Unit conversion of cumulative production in million short tons to TWhr

NuclearPower

This sheet calculates representative, low and high estimates of nuclear power direct footprint. Below is the description for each section:

Rows 3- 12: Estimates of energy production provided by EIA.

Rows 13-23: Cumulative production calculation

Rows 19-25: According to the EIA, these are the nameplate capacity factor estimates.

Row 28: The footprint, linked to FootprintEstimates worksheet for mining activities.

Area (ha/billion Kwhr) = Footprint (ha/billion Kwhr)*EIA Nuclear Production

(billion Kwhr)* % Domestic production

Row 29: According to the EIA, the proportion of domestic mining operations.

Rows 43-56: The estimated area of impact for cumulative production of Nuclear power

for waste storage

Rows 57-81: The estimated area of impact for Nuclear power plant sites (ha)

Rows 85-110: The calculations to convert impact (ha/billion KWhr) and energy

production into consistent units (km2/TWhr)

Row 113-125: Calculation of cumulative production in TWhr

*BiofuelsImpact*

This sheet calculates representative, low and high estimates of biofuels footprint. Below is the description for each section:

Rows 1- 28: Estimates of energy production provided by EIA.

Rows 31-34: Based on EIA, the proportion of domestic ethanol from corn, cellulose and other feedstocks

Rows 40-48: We determined the maximum production between 2012 to 2040 If forecasted peak production was less or equal to production in 2011 then there is no added foot print. When forecasted peak production was greater than 2011 production, new biofuel production was the difference between 2011 production and the forecasted peak production.

Rows 51-58: The footprint, linked to FootprintEstimates worksheet for each source.

Rows 61-107: Estimated new area required for biofuels.

New Area (acres) = (1000000*Newbiofuel Production (million barrels/day)*% domestic Production*365.25 days*42)/footprint (gallons/acre/year)

Rows 108-116: estimated percent attributable to coproducts

Rows 121-133: Estimated new area required for biofuels, adjusted by removing land attributable to co-products.

Rows 166-189: Incorporating the estimated area of impact for peak production from acres of crop producing gallons of product with the estimated energy output (e.g., BTUs per gallon). These conversions provide the comparable units of km^2^/TWhr.

Row 199-227: Based on EIA production estimates, this calculates the annual biofuels

consumption out to 2040

BiomassPower

This sheet calculates representative, low and high estimates of biomass footprint. Below is the description for each section:

Rows 1-4: Estimates of energy production provided by EIA.

Rows 6-8: We determined the maximum production from 2012 to 2040 as the peak

production. If forecasted peak production is less or equal to previous production then there is no added foot print. When forecasted peak production is greater than previous production, new renewable energy production is the difference between previous production and the forecasted peak production.

Rows 10-18: Nameplate capacity factor estimates from EIA.

Rows 20-22: The footprint, linked to FootprintEstimates worksheet for each source.

Rows 24-35: Estimated new area required for biofuels. New Area (miles^2^) = footprint (miles^2^/GW plant) * Nameplate capacity increase (gigawatt)

Rows 36-42: The calculations to convert impact and energy production into consistent units (km2/TWhr) for each scenarios

Row 44-51: Based on EIA production estimates, this calculates the annual biomass consumption out to 2040

OtherRenewableEnergy

This sheet focuses on representative, low and high estimates of remaining renewable energy footprint. Below is the description for each section:

Rows 1- 12: Estimates of energy production provided by EIA.

Rows 14-24: We determined the maximum production from 2012 to 2040 as the peak

production. If forecasted peak production is less or equal to previous production then there is no added foot print. When forecasted peak production is greater than previous production, new renewable energy production is the difference between previous production and the forecasted peak production.

Rows 26-50: Nameplate capacity factor estimates from EIA.

Rows 54-106: The footprint, linked to FootprintEstimates worksheet for each source.

Rows 110-121: Estimated new area required for renewable energy and calculations to convert impact and energy production into consistent units (km2/TWhr) for each scenarios.

Energy Sprawl (km^2^/Thhr/yr)=

[Area Required for new capacity(km^2^/GW)*(365.25*24*Nameplate Capacity Factor)]*1000

Row 137-147: Based on EIA production estimates, this calculates the annual Renewable energy consumption out to 2040

ConvNG_AK_wells

EIA breaks out wells for different regions. This sheet focuses solely on conventional Natural gas wells for Alaska. For each scenario, we used the estimated well lifetime (cell F8), we divided the total annual production by the number of producing wells for each year between 1995 and 2005 to estimate the average gross withdrawals per well (cell G8), and ratio of dry gas to wet gas (cell J10) to adjust production value (Cell H12) to calculate the number of new wells required to achieve projected production levels each year. Based on these values, we estimated the number of new wells required to meet production out to 2040 (cell G54 for the Reference Scenario). For both conventional and unconventional sources, well abandonment rates were assumed to be the inverse of well lifetime (column E)

*ConvNG_Lower48_wells*

EIA breaks out wells for different regions. This sheet focuses solely on conventional Natural gas wells for the lower 48 states. For each scenario, we first estimated well lifetime (cell I3). Next we divided the total annual production by the number of producing wells for each year between 1995 and 2005 average gross withdrawals per well (cell J3), and ratio of dry gas to wet gas (cell L5) to adjust production value (Cell J7) to calculate the number of new wells required to achieve projected production levels each year. We then totaled the number of new wells required to meet production out to 2040 (cell J49 for the Reference Scenario). For both conventional and unconventional sources, well abandonment rates were assumed to be the inverse of well lifetime (column H).

*Shale_wells*

This sheet focuses solely on natural gas wells using shale gas wells. Projected production was an average of EIA estimates across all unconventional plays (similar oil and gas accumulations sharing geologic and geographic properties), weighted by the recoverable resource in each play. We used technically recoverable estimates for each play to get average well spacing, an estimated average estimated ultimate recovery (EUR), and technically recoverable amount of resource to obtain a weighted average for EUR across the country (cell G140).

For each scenario, we used the estimated well lifetime (cell F92), average EUR/well (cell G92) converted to EUR (Mcf/well/year) (cell I94), ratio of dry gas to wet gas (cell J97) to adjust production value (Cell H99) to estimate the number of new wells required to meet production out to 2040 (cell J49 for the Reference Scenario).

*TightGas_wells*

This sheet focuses solely on natural gas wells using tight gas technology. Projected production was an average of EIA estimates across all unconventional plays (similar oil and gas accumulations sharing geologic and geographic properties), weighted by the recoverable resource in each play. We used technically recoverable estimates for each play to get average well spacing, an estimated average estimated ultimate recovery (EUR), and technically recoverable amount of resource to obtain a weighted average for EUR across the country (cell M88).

For each scenario, we used the estimated well lifetime (cell F93), average EUR/well (cell G93) converted to EUR (Mcf/well/year) (cell I94), ratio of dry gas to wet gas (cell J98) to adjust production value (Cell H100) to estimate the number of new wells required to meet production out to 2040 (cell G141 for the Reference Scenario).

*CBM_wells*

This sheet focuses solely on natural gas wells from coalbed methane sources. Projected production was an average of EIA estimates across all unconventional plays (similar oil and gas accumulations sharing geologic and geographic properties), weighted by the recoverable resource in each play. We used technically recoverable estimates for each play to get average well spacing, an estimated average estimated ultimate recovery (EUR), and technically recoverable amount of resource to obtain a weighted average for EUR across the country (cell L38).

For each scenario, we used the estimated well lifetime (cell 45), average EUR/well (cell G45) converted to EUR (Mcf/well/year) (cell H47), ratio of dry gas to wet gas (cell J50) to adjust production value (Cell H51) to estimate the number of new wells required to meet production out to 2040 (cell G93 for the Reference Scenario).

ConvOil_AK_wells

This sheet focuses solely on conventional oil wells for lower 48 states. For each scenario, we used the estimated well lifetime (cell G4), we divided the total annual production by the number of producing wells for each year between 1995 and 2005 (cell H3) to calculate the number of new wells required to achieve projected production levels each year. We then totaled the number of new wells required to meet production out to 2040 (cell H47 for the Reference Scenario). For both conventional and unconventional sources, well abandonment rates were assumed to be the inverse of well lifetime (column F)

ConvOilL48_wells

EIA breaks out wells for different regions. This sheet focuses solely on conventional Natural gas wells for the lower 48 states. For each scenario, we used the estimated well lifetime (cell I3), we divided the total annual production by the number of producing wells for each year between 1995 and 2005 average gross withdrawals per well (cell J3), and ratio of dry gas to wet gas (cell L5) to adjust production value (Cell J7) to calculate the number of new wells required to achieve projected production levels each year. We then totaled the number of new wells required to meet production out to 2040 (cell J49 for the Reference Scenario). For both conventional and unconventional sources, well abandonment rates were assumed to be the inverse of well lifetime (column F)

TightOil_wells

This sheet focuses solely on natural gas wells using tight oil production. We used technically recoverable estimates for each play to get average well spacing, an estimated average estimated ultimate recovery (EUR), and technically recoverable amount of resource to obtain a weighted average for EUR across the country (cell M88). We divided the total annual production by the number of producing wells for each year between 1995 and 2005.

For each scenario, we used the estimated well lifetime (cell G96), average EUR/well (cell K96) converted to EUR (Mcf/well/year) (cell K97) to calculate the number of new wells required to achieve projected production levels each year. We then totaled the number of new wells required to meet production out to 2040 (cell H140 for the Reference Scenario). For both conventional and unconventional sources, well abandonment rates were assumed to be the inverse of well lifetime (column F).

Comparison_wells

For each scenario, EIA estimates the total number of wells drilled each year, but does not specify how many of these wells are attributable to each type of production. To address this we used the above sheets to estimate the number of new wells that would be required to meet EIA’s projected production *for each energy type*. We then used these values to estimate the proportion of wells attributable to each type of well (Column L rows 3-12 for the Reference Scenario). To get a final estimate of the number of wells drilled for each type by 2040 (Column N rows 3-12 for the Reference Scenario), we used EIA’s total well numbers times this proportion attributable to each well type (Column M rows 3-12).

Comparison_Bracket

This sheet was developed to evaluate the sensitivity of our assumptions on the mixture of energy sources for the wells. With the same set up as the Comparison sheet, we evaluated the footprint from all drilling, assuming all wells belonged to a single type.

Conv.NG

This sheet calculates representative, low and high energy sprawl estimates for conventional natural gas (on shore Alaska and lower-48 states). Below is the description for each section:

Rows 4-10: Estimates of energy production provided by EIA.

Rows 13-25: Cumulative production from 2012 to 2040 for the lower 48 wells.

Rows 27-39: Cumulative production from 2012 to 2040 for conventional, on-shore wells in Alaska.

Rows 36-65 focus on the lower estimated impact (i.e., small well pads).

Row 48: Refer back to the “Comparison” Worksheet for estimated number of wells

Row 49: The number of wells per well pad for conventional natural gas production.

Row 50: Refers to the direct footprint of the well pad multiplied by the number of well pads

Row 51: Refers to the footprint of additional features associated with drilling footprint multiplied by the number of well pads

Rows 53-57 repeats above steps for the Alaska wells.

Row 62: Total area impacted at the well pad level

Row 66: Landscape level impacted by accounting for spacing requirements.

Row 71: Energy sprawl estimated at the well pad level

Row 74: Energy sprawl estimated at landscape level impacted.

Rows 82-109: focus on the representative estimated impact

Rows 110-143: focus on the broader estimated impact (i.e., larger well pads).

ShaleGas

This sheet focuses on representative, compact and broad energy sprawl estimates for shale gas. Below is the description for each section:

Rows 1- 4: Estimates of cumulative energy production provided by EIA.

Rows 9-20: We determined the cumulative production from 2012 to 2040.

Rows 23-47: focus on the lower estimated impact (i.e., small well pads).

Row 26: Refer back to the “Comparison” Worksheet for estimated number of wells

Row 27: The number of wells per well pad for shale gas production.

Row 28: Refers to the direct footprint of the well pad multiplied by the number of well pads

Row 29: Refers to the footprint of additional features associated with drilling footprint multiplied by the number of well pads

Row 34: Total area impacted at the well pad level

Row 39: Landscape level impacted by accounting for spacing requirements.

Row 44: Energy sprawl estimated at the well pad level

Row 47: Energy sprawl estimated at landscape level impacted.

Rows 55-80: focus on the representative estimated impact

Rows 86-109: focus on the broader estimated impact (i.e., larger well pads).

Row 113-116: Based on EIA production estimates, this calculates the annual energy consumption out to 2040

TightGas

This sheet focuses on representative, compact and broad energy sprawl estimates for tight gas. Below is the description for each section:

Rows 1- 5: Estimates of cumulative energy production provided by EIA.

Rows 9-19: Cumulative production from 2012 to 2040.

Rows 23-47: Focus on the lower estimated impact (i.e., small well pads).

Row 26: Refer back to the “Comparison” Worksheet for estimated number of wells

Row 27: The number of wells per well pad for tight gas production.

Row 28: Refers to the direct footprint of the well pad multiplied by the number of well pads

Row 29: Refers to the footprint of additional features associated with drilling footprint multiplied by the number of well pads

Row 34: Total area impacted at the well pad level

Row 39: Landscape level impacted by accounting for spacing requirements.

Row 44: Energy sprawl estimated at the well pad level

Row 47: Energy sprawl estimated at landscape level impacted.

Rows 55-80: focus on the representative estimated impact

Rows 81-105: focus on the high estimated impact (i.e., larger well pads).

Row 110-114: Based on EIA production estimates, this calculates the annual energy consumption out to 2040

CoalbedMethane

This sheet focuses on representative, compact and broad energy sprawl estimates for coalbed methane. Below is the description for each section:

Rows 1- 5: Estimates of cumulative energy production provided by EIA.

Rows 9-19: We determined the cumulative production from 2012 to 2040.

Rows 23-47: focus on the lower estimated impact (i.e., small well pads).

Row 25: Refer back to the “Comparison” Worksheet for estimated number of wells

Row 26: The number of wells per well pad for coalbed methane production.

Row 27: Refers to the direct footprint of the well pad multiplied by the number of well pads

Row 28: Refers to the footprint of additional features associated with drilling footprint multiplied by the number of well pads

Row 33: Total area impacted at the well pad level

Row 38: Landscape level impacted by accounting for spacing requirements.

Row 43: Energy sprawl estimated at the well pad level

Row 46: Energy sprawl estimated at landscape level impacted.

Rows 51-75: focus on the representative estimated impact

Rows 83-107: focus on the broader estimated impact (i.e., larger well pads).

Row 110-114: Based on EIA production estimates, this calculates the annual energy consumption out to 2040

Conv.Oil

This sheet focuses on representative, compact and broad energy sprawl estimates for conventional oil (on shore Alaska and lower-48 states) Below is the description for each section:

Rows 1- 10: Estimates of energy production provided by EIA.

Rows 13-25: We determined the cumulative production from 2012 to 2040 for the on-shore Alaska wells.

Rows 26-39: We determined the cumulative production from 2012 to 2040 for the conventional wells in the lower 48 states.

Rows 36-65 focus on the lower estimated impact (i.e., small well pads).

Row 39: Refer back to the “Comparison” Worksheet for estimated number of

wells

Row 40: The number of wells per well pad for conventional natural gas

production.

Row 41: Refers to the direct footprint of the well pad multiplied by the number

of well pads

Row 42: Refers to the footprint of additional features associated with drilling

footprint multiplied by the number of well pads

Row 52: Total area impacted at the well pad level

Row 57: Landscape level impacted by accounting for spacing requirements.

Row 62: Energy sprawl estimated at the well pad level

Row 65: Energy sprawl estimated at landscape level impacted.

Rows 72-102 focus on the representative estimated impact

Rows 109-138 focus on the broader estimated impact (i.e., larger well pads).

*TightOil*

This sheet focuses on representative, compact and broad energy sprawl estimates for tight oil (on shore Alaska and lower-48 states) Below is the description for each section:

Rows 1- 7: Estimates of cumulative energy production provided by EIA.

Rows 10-22: We determined the cumulative production from 2012 to 2040 for the on- shore Alaska wells.

Rows 25-49 focus on the lower estimated impact (i.e., small well pads).

Row 28: Refer back to the “Comparison” Worksheet for estimated number of

wells

Row 29: The number of wells per well pad for conventional natural gas

production.

Row 30: Refers to the direct footprint of the well pad multiplied by the number

of well pads

Row 31: Refers to the footprint of additional features associated with drilling

footprint multiplied by the number of well pads

Row 36: Total area impacted at the well pad level

Row 41: Landscape level impacted by accounting for spacing requirements.

Row 46: Energy sprawl estimated at the well pad level

Row 49: Energy sprawl estimated at landscape level impacted.

Rows 52-76: focus on the representative estimated impact

Rows 81-105 focus on the broader estimated impact (i.e., larger well pads).

Row 108-111: Based on EIA production estimates, this calculates the annual energy consumption out to 2040

PropEngyProd

This sheet summarizes the total amount of energy produced in TWhr for each sector and scenario according to EIA.

Energy Sprawl_in2040

This is a summary table that pulls together all the energy sprawl estimates (km^2^/TWhr) for each energy source.

Rows 1-33: summarize direct footprint (i.e., no spacing)

Rows 42-80: summarize the landscape level impact (i.e., spacing requirements)

MyConsumptionNumbers

This worksheet aggregates each sector’s estimated consumption values to provide an estimated effency gains.

Catchup

This worksheet compares the energy sprawl values across sectors.

SummaryImpact

This sheet summarizes the total direct footprint and the landscape level impact by each sector for each scenario.

SummaryImpact_graphic

This sheet is linked to SummaryImpact worksheet. It is a more organized worksheet with the graphics provided.

Rows 3-20: Direct Impact

Columns C-G are compact impact

Columns I-M are representative Impact

Columns P-V are Broad Impact

Rows 24-29: Landscape level impact due to spacing requirements.

EnergyIntensity

Refers to EIA’s energy consumption for each sector and scenario.
