# Supplementary material for: Energy Sprawl Is the Largest Driver of Land Use Change in United States
Source: PLoS One. 2016 Sep 8;11(9):e0162269. doi: 10.1371/journal.pone.0162269 (PMC5015902; doi:10.1371/journal.pone.0162269)
Supplement: S1 Table — (DOCX) [file pone.0162269.s003.docx]

**Supplement1 Table. Estimated footprint and citations used to estimate the compact, representative, and broad spatial impact for each energy source.**

|  |  | |  |  | Compact | | Representative | | Extensive | |  |
| --- | --- | --- | --- | --- | --- | --- | --- | --- | --- | --- | --- |
| Type | Sector | | Energy Source | Units | Estimate | Citation | Estimate | Citation | Estimate | Citation | Comment |
| Renewable | Biomass | |  | Miles^2^/GW plant | 1257.0 |  | 1824.3 |  | 2825.3 |  | Supplemental Table 1 References |
|  | Biofuels | | Corn | Gallons/acre per year | 440.1 | [1,2] | 481.8 | [1,2] | 591.6 | [1,2] |  |
|  |  | | Cellulose | Gallons/acre per year | 1411.0 | [3,4] | 313.6 | [1] | 214.6 | [1] |  |
|  |  | | Other Feed stocks (aka sugar cane) | Gallons/acre per year | 773.5 | [5–7] | 646 | [5–7] | 518.4 | [5–7] | Based on Brazil yields |
|  |  | | Biodiesel rates | Gallons/acre per year | 156.9 | [2] | 124.9 | [2] | 117.9 | [2] |  |
|  |  | | Liquids from biomass rates | Gallons/acre per year | 1087 | [6,8] | 936 | [6,8] | 785 | [6,8] |  |
|  | Geothermal | |  | Km^2^/GW plant | 16.2 | [9] | 38.8 | [9] | 82.9 | [9] |  |
|  | Solar Thermal | |  | Km^2^/GW plant | 26.2 | [10] | 38.85 | [10] | 56.5 | [10] |  |
|  | Solar Photovoltaic | |  | Km^2^/GW plant | 28.2 | [10] | 34.4 | [10] | 38.9 | [10] |  |
|  | Wind | | Direct Impact | Km^2^/GW plant | 1 | [9] | 3.8 | [10] | 4 | [9] |  |
|  |  | | Landscape Level Impact | Km^2^/GW plant | 202.3 | [10] | 368.3 | [10] | 465.3 | [10] |  |
|  | Hydroelectric | | | Km^2^/GW plant | 23.9 | [11,12] | 62.4 | [11,12] | 321.9 | [11,12] |  |
| Nuclear | Mining Impacts | | | Hectares/billion KWhr of plant operation | 5.05 | [13] | 34.8 | Midpoint | 64.6 | [13] |  |
|  | nuclear waste storage impacts | | | Hectares/billion KWhr of plant operation | 0.25 | [13] | 0.29 | Midpoint | 0.32 | [13] |  |
|  | nuclear plant site impacts | | | Hectares/GW of new Capacity | 250 | [14] | 325 | Midpoint | 400 | [14] |  |
| Fossil Fuel | Natural Gas | Conventional | | Acres | 4.8 | Hall Sawyer (unpublished data) | 6.6 | [15] | 8.95 | [15] |  |
|  |  | Landscape level impact | | Acres |  |  | 33 | [16,17] |  |  |  |
|  |  | Tight Gas | | Acres | 10 | [18,19] | 14 | [18,19] | 32.5 | [18,19] |  |
|  |  | Shale Gas | | Acres | 5.74 | [20] | 6.5 | [21] | 9.8 | [15] |  |
|  |  | Coalbed Methane | | Acres | 3.7 | [22] | 4.98 | [23] | 10 | Same reference as compact |  |
|  | Oil | Conventional | | Acres | 4.8 | Hall Sawyer (unpublished data) | 6.6 | [15] | 8.95 | [15] |  |
|  |  | Tight Oil | | Acres | 5.74 | [20] | 6.5 | [21] | 9.8 | [15] |  |
|  | Coal | Underground | | Hectares/million short tons | 46 | [24,25] | 118 | [24,25] | 281 | [24,25] |  |
|  |  | Above Ground (Appalachian Region | | Hectares/million short tons | 40.5 | [26] | 212 | [27] | 337 | [28] |  |
|  |  | Above Ground (Interior) | | Hectares/million short tons | 497 | [28] | 497 | [27] | 1860 | [29,30] |  |
|  |  | Above Ground (West) | | Hectares/million short tons | 328 | [25,30,31] | 515 | [25,30,31] | 828 | [25,30,31] |  |
|  |  | Power Plant | | Hectares/million short tons |  |  | 0.25 | [27] |  |  |  |

**References**

1. Wang M, Huo H, Arora S. Methods of dealing with co-products of biofuels in life-cycle analysis and consequent results within the U.S. context. Energy Policy. Elsevier; 2011;39: 5726–5736. doi:10.1016/j.enpol.2010.03.052

2. United States Department of Agriculture. USDA Agricultural Projections to 2023. Long-term Projections Report OCE-2014-1; 2014.

3. Heaton EA, Dohleman FG, Long SP. Meeting US biofuel goals with less land: the potential of Miscanthus. Glob Chang Biol. 2008;14: 2000–2014. doi:10.1111/j.1365-2486.2008.01662.x

4. Lynd LR, Laser MS, Brandsby D, Dale BE, Davison B. How biotech can transform biofuels. Nat Biotechnol. 2008;26: 169–172.

5. Macedo IC, Seabra JEA, Sivla JEA. Greenhouse gas emissions in the production and use of ethanol from sugarcane in Brazil: The 2005/2006 averages and a prediction for 2020. Biomass and Bioenergy. 2008;32: 582–595.

6. United States Energy Information Administration. Average Price (Cents/kilowatthour) by State by Provider, 1990-2014. 2014. Available: https://www.eia.gov/electricity/data/state/

7. Food and Agriculture Organization of the United Nations. Production of commodity in selected country. In: 2013. Available: http://faostat3.fao.org

8. Phillips S, Aden A, Jechura J, Dayton D, Eggeman T. Thermochemical Ethanol via Indirect Gasification and Mixed Alcohol Synthesis of Lignocellulosic Biomass. Golden, CO, USA; 2007.

9. Ong S, Campbell C, Heath GA. Land Use for Wind, Solar, and Geothermal Electricity Generation Facilities in the United States. 2012.

10. Ong S, Campbell C, Denholm P, Margolis R, Heath G. Land-Use Requirements for Solar Power Plants in the United States Land-Use. Golden, CO; 2013.

11. Federal Energy Regulatory Commission. Federal Energy Regulatory Commission (FERC) . Available: www.ferc.gov/industries/ hydropower/gen-info/licensing/hydrokinetics.asp

12. United States Geological Survey. National Hydrography Dataset (NHD) . Available: http://nhd.usgs.gov/index.html

13. Spitzley D V., Keoleian GA. Life cycle environmental and economic assessment of willow biomass electricity: a comparison with other renewable and non- renewable sources. 2005.

14. Mayeda P, Riener K. Economic Benefits of Diablo Canyon Power Plant. 2013.

15. BLM. Reasonably Foreseeable Development Scenario for Fluid Minerals. Jackson, MS 39206; 2008.

16. Colorado Oil and Gas Conservation Commission. Series drilling, developement, production and abandonment . 2015 [cited 14 Feb 2015] pp. 1–70. Available: https://cogcc.state.co.us/reg.html#/rules

17. Texas Office of the Secretary of State. RULE §3.37: Statewide Spacing Rule . 2004 [cited 14 Feb 2015]. Available: http://texreg.sos.state.tx.us

18. Amos J. Measuring the Direct Landscape Impact of Natural Gas Drilling . 2009. Available: http://blog.skytruth.org/2009/12/measuring-direct-landscape-impact-of.html

19. Jonah field and pinedale anticline natural gas success story . Available: http://www.wyohistory.org/essays/jonah-field-and-pinedale-anticline-natural-gas-success-story?page=2

20. Office C. New York State Department of Environmental Conservation. 2011;

21. Gottschalk K, Service UF, Benham B, Tech V, Chambers R, William C. Exploring the Environmental Effects of Shale Gas Development in the Chesapeake Bay Watershed. 2012.

22. Bureau of Land Mangament. Approved Pinedale Resource Management Plan: Appendix 10. Pinedale, Wyoming; 2008.

23. Fisher JB. No Title. In: 18th Internatuional Low Rank Fuels Symposium . 2003. Available: (http://ipec.utulsa.edu/Conf2001/fisher_92.pdf

24. Fthenakis V, Kim HC. Land use and electricity generation: A life-cycle analysis. Renew Sustain Energy Rev. 2009;13: 1465–1474. doi:10.1016/j.rser.2008.09.017

25. State of Colorado. Colorado Coal Mines . Available: http://mining.state.co.us/SiteCollectionDocuments/ColoradoCoalMines.pdf

26. US Environmental Protection Agency. Draft programmatic environmental impact statement on mountaintop mining/valley fills in Appalachia. 2003.

27. Fthenakis V, Kim HC. Land use and electricity generation: A life-cycle analysis. Renew Sustain Energy Rev. 2009;13: 1465–1474. doi:10.1016/j.rser.2008.09.017

28. Robeck KE, Ballou SW, South DW, Davis M, Chiu SY, Baker JE, et al. Land Use and Energy. Argonne, Illinois; 1980.

29. Existing coal mines in Texas. Available: http://www.sourcewatch.org/index.php/Category:Existing_coal_mines_in_Texas

30. Jewett Mine. Available: http://www.sourcewatch.org/index.php/Jewett_Mine

31. State of North Dakota.. Available: http://psc.nd.gov/jurisdiction/coalmining/companies.php
