# Supplementary material for: Energy Sprawl Is the Largest Driver of Land Use Change in United States
Source: PLoS One. 2016 Sep 8;11(9):e0162269. doi: 10.1371/journal.pone.0162269 (PMC5015902; doi:10.1371/journal.pone.0162269)
Supplement: S2 Table — (DOCX) [file pone.0162269.s004.docx]

**S2 Table. List of experimental studies used to estimate the representative spatial impact of “other biomass used to produce any liquid fuel”.**

| Citation | Region | Mg/ha | tons/acre |
| --- | --- | --- | --- |
| Parrish and Fike (2005)[1] |  | 15 | 6.07 |
| Smeets et al. (2009)[2] |  | 20 | 8.10 |
| Dunn et al. (2011)[3] |  | 14.7 | 5.95 |
| U.S. Billion-Ton Update: Biomass Supply for a Bioenergy and Bioproducts Industry[4] |  | 12.4 | 5.00 |
|  |  | 18.3 | 7.40 |
|  |  | 23.2 | 9.40 |
| Guretzky et al. (2011) [5] | Oklahoma | 10.4 | 4.21 |
|  | Oklahoma | 14 | 5.67 |
|  | Oklahoma | 13.1 | 5.30 |
|  | Oklahoma | 15 | 6.07 |
|  | Oklahoma | 13.7 | 5.55 |
|  | Oklahoma | 15.5 | 6.28 |
|  | Oklahoma | 10.8 | 4.37 |
|  | Oklahoma | 11.7 | 4.74 |
|  | Oklahoma | 13.3 | 5.38 |
|  | Oklahoma | 14.4 | 5.83 |
|  | Oklahoma | 13.7 | 5.55 |
|  | Oklahoma | 14.6 | 5.91 |
|  | Oklahoma | 12.2 | 4.94 |
|  | Oklahoma | 15.5 | 6.28 |
|  | Oklahoma | 18.4 | 7.45 |
|  | Oklahoma | 18 | 7.29 |
|  | Oklahoma | 22.8 | 9.23 |
|  | Oklahoma | 21 | 8.50 |
| (Muir et al. 2001)[6] | Texas | 9.2 | 3.72 |
|  | Texas | 9.6 | 3.89 |
|  | Texas | 9.6 | 3.89 |
|  | Texas | 10.4 | 4.21 |
|  | Texas | 10.5 | 4.25 |
|  | Texas | 10.7 | 4.33 |
|  | Texas | 10.8 | 4.37 |
|  | Texas | 12.3 | 4.98 |
|  | Texas | 3.9 | 1.58 |
|  | Texas | 6.7 | 2.71 |
|  | Texas | 8.8 | 3.56 |
|  | Texas | 6.6 | 2.67 |
|  | Texas | 15.7 | 6.36 |
|  | Texas | 16.7 | 6.76 |
|  | Texas | 7.9 | 3.20 |
|  | Texas | 8.4 | 3.40 |
|  | Texas | 12.2 | 4.94 |
|  | Texas | 10 | 4.05 |
|  | Texas | 12.1 | 4.90 |
|  | Texas | 15.9 | 6.44 |
| Cassida et al. (2005) [7] | Texas, Arkansas, and Louisiana | 15 | 6.07 |
| Schmer et al. (2008)[8] | Nebraska | 5.2 | 2.11 |
|  | Nebraska | 3.9 | 1.58 |
|  | Nebraska | 4.9 | 1.98 |
|  | Nebraska | 4.8 | 1.94 |
|  | South Dakota | 8 | 3.24 |
|  | South Dakota | 6.6 | 2.67 |
|  | South Dakota | 8.4 | 3.40 |
|  | South Dakota | 9.9 | 4.01 |
|  | North Dakota | 5 | 2.02 |
|  | North Dakota | 8.2 | 3.32 |
|  | Nebraska | 7.1 | 2.87 |
|  | Nebraska | 8.8 | 3.56 |
|  | Nebraska | 5.5 | 2.23 |
|  | Nebraska | 7.2 | 2.91 |
|  | South Dakota | 6.9 | 2.79 |
|  | South Dakota | 10.5 | 4.25 |
|  | South Dakota | 8.3 | 3.36 |
|  | South Dakota | 11.4 | 4.62 |
|  | North Dakota | 8.3 | 3.36 |
|  | North Dakota | 8.4 | 3.40 |
|  | Nebraska | 6.2 | 2.51 |
|  | Nebraska | 7.5 | 3.04 |
|  | Nebraska | 6.3 | 2.55 |
|  | South Dakota | 6.1 | 2.47 |
|  | South Dakota | 5.5 | 2.23 |
|  | South Dakota | 3.7 | 1.50 |
|  | South Dakota | 12.1 | 4.90 |
|  | North Dakota | 6.1 | 2.47 |
|  | North Dakota | 6.9 | 2.79 |

**References**

1. Parrish DJ, Fike JH. The Biology and Agronomy of Switchgrass for Biofuels. CRC Crit Rev Plant Sci. 2005;24: 423–459. doi:10.1080/07352680500316433

2. Smeets EMW, Lewandowski IM, Faaij APC. The economical and environmental performance of miscanthus and switchgrass production and supply chains in a European setting. Renew Sustain Energy Rev. 2009;13: 1230–1245. doi:10.1016/j.rser.2008.09.006

3. Dunn JB, Eason J, Wang MQ. Updated Sugarcane and Switchgrass Parameters in the GREET Model. 2011.

4. U.S. Department of Energy. U.S. Billion-Ton Update: Biomass Supply for a Bioenergy and Bioproducts Industry. Oak Ridge, Tennessee; 2011.

5. Guretzky JA, Biermacher JT, Cook BJ, Kering MK, Mosali J. Switchgrass for forage and bioenergy: Harvest and nitrogen rate effects on biomass yields and nutrient composition. Plant Soil. 2010;339: 69–81. doi:10.1007/s11104-010-0376-4

6. Muir JP, Sanderson MA, Ocumpaugh WR, Jones RM, Reed RL. Biomass production of “Alamo” switchgrass in response to nitrogen, phosphorus, and row spacing. Agron J. 2001;93: 896–901.

7. Cassida KA, Muir JP, Hussey MA, Read JC, Vento BC, Ocumpaugh WR. Biomass yield and stand charac- teristics of switchgrass in south central U.S. environments. Crop Sci. 2005;45: 673–681.

8. Schmer MR, Vogel KP, Mitchell RB, Perrin RK. Net energy of cellulosic ethanol from switchgrass. Proc Natl Acad Sci U S A. 2008;105: 464–9. doi:10.1073/pnas.0704767105
